# Association study of six SNPs in PRM1, PRM2 and TNP2 genes in iranian infertile men with idiopathic azoospermia

**Published:** 2012-07

**Authors:** Elham Siasi, Ahmad Aleyasin, Javad Mowla, Hamid Sahebkashaf

**Affiliations:** 1 *Department of Genetic, Tarbiat Modares University, Tehran, Iran. *; 2 *National Institute for Genetic Engineering and Biotechnology, Tehran, Iran. *; 3 *Navide Institute of Infertility, Tehran, Iran. *

**Keywords:** *Male infertility*, *SNP*, *PRM1gene*, *PRM2 gene*, *TNP2 gene*

## Abstract

**Background:** Histones are replaced by protamines to condensate and package DNA into the sperm head during mammalian spermatogenesis. Protamine genes defects have been reported to cause sperm DNA damage and male infertility.

**Objective:** In this study relationship among some protamines genes family SNPs include PRM1 (C321A), PRM2 (C248T) and TNP2 (T1019C), (G1272C), (G del in 1036 and 1046 bp) were studied in 96 idiopathic infertile men with azoospermia or oligospermia and 100 normal control men.

**Materials and Methods:** Analysis of SNPs was performed using restriction fragment length polymorphism (PCR-RFLP), single strand conformational polymorphism (PCR-SSCP) and PCR sequencing.

**Results:** No polymorphisms were found for tested SNPs except for PRM1 (C321A) and TNP2 (G1272C) in which frequency of altered AA and GG genotypes were slightly higher in infertile case group. Statistical analysis showed no significant association related to PRM1 (C321A) p=0.805 and TNP2 (G1272C) loci p=0.654.

**Conclusion:** These results are consistent with previous studies and indicating that all tested SNPs was not associated with oligospermia and azospermia and idiopatic male infertility in Iranian population.

## Introduction

Approximately 15-17% of couples who are attempting to conceive are infertile due to male infertility. More than 90% of male infertility is due to poor sperm quality. Idiopatic male infertility is a subgroup of infertile oliogospermic or azospermic males with unknown causes which is often associated with genetic and epigenetic abnormalities (-). Mutation in protamine genes have been reported to cause abnormal spermatogenesis and defect in imprinting and induce sperm chromatin damage and DNA breaks (1, 2, 5, 6).

**Figure 1:**
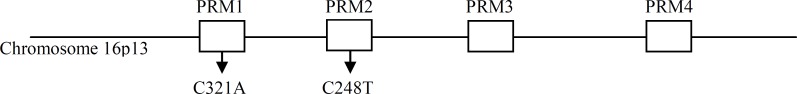
Schematic representation of protamine genes in multigenic locus on chromosome 16p13 with position of SNPs in PRM1 and PRM2 genes    (33) .

The nucleoprotein genes PRM1, PRM2 and TNP2 are closely linked in a stretch of DNA, 13-15 kb long on human chromosome 16p13.3 that are categorized in protamine gene family (Figure 1) (-). Protamines are the major DNA binding proteins in the sperm nucleus that cause DNA condensation and packaging in spermatozoa by histones replacement during spermatogenesis. The structure of chromatin become permanently remodeling undergo complex morphologic, physiologic and biochemical modifications. 

In the late elongation stage of spermatids development, transition proteins are replaced by protamines (4, 8, -). It appears that protamines stabilize sperm chromatin by their assembly in the minor groove of DNA into densely packed arrays linked by intermolecular and intramolecular disulfide bonds. After this binding substitution the process of gene transcription is completely inactivated and result in formation of mature spermatozoa (12, 14). Mutation in protamine genes has been reported to be associated with sperm penetration dysfunction, failure of embryonic development and DNA sperm damage (14, 17). These sperm DNA damage and induction of apoptotic pathway induce sperm counts reduction and motility (2, 18, 19). 

Protamine defected proteins cause abnormal condensation of sperm chromatin, increases sperm DNA strand breaks and immobility of spermatozoa that can led to male infertility (2, 12, 14, 20). Another important gene that play role in chromatin condensation is transition nuclear protein 2 (TNP2) that are replaced by the protamines PRM1 and PRM2 during chromatin condensation in the mature sperm nucleus (11, 12, 15, 16, 21). TNP2 is required in the establishment of species specific morphology of sperm DNA and changes of the nuclear shaping (-).

Defects in TNP2 proteins lead to abnormalities in sperm head due to acrosomal defects, impairing migration of the spermatozoa through the female genital tract and inability of the spermatozoa to penetrate the zonapellucida that cause male infertility (25, 26). Recent investigations represented different variations in PRM1, PRM2 and TNP2 gene sequences in human with various relationships to male infertility (1, 2, 4, 9, 18, 28, 29). In this study, six SNPs in PRM1 (C321A), PRM2 (C248T) and TNP2 (T1019C), (G1272C) and G deletion in nucleotides 1036 and 1046 have been examined in idiopathic infertile men with oligospermia and azoospermia and normal individuals to analyze their relationships with idiopathic male infertility in Iranian cases. 

## Materials and methods

**Sampling**

Patient and control groups were recruited from Navid infertility center, Tehran-Iran. Where they are referred for male infertility included individuals of Iranian origin at the same ages. Infertile patients included in this study were seeking a complete andrological diagnostic work-up for couple infertility. 

All infertile patients were defined as ‘idiopathic’ and selected on the basis of a comprehensive andrological examination including medical history and physical examination, semen analysis, scrotal ultrasound, hormone analysis and karyotype screening. Patients with mono or bilateral cryptorchidism, varicocele, previous testis trauma, obstructive azoospermia, recurrent infections, iatrogenic infertility, hypogonadotrophic hypogonadism, karyotype anomalies were excluded. 

Cases group were contained of 19 idiopathic severe oligozoospermic individuals with <5 million sperm/ml and 77 idiopathic azoospermic individuals with no detectable sperm in the semen. The normal control group consisted of 100 normospermic male couple with >20 million sperm/ml in their semen at the same ages with case group. A detailed medical history and physical examination were performed for the investigated control and all were fertile and had normal child.

**Analysis of the PRM1, PRM2 and TNP2 genes**

DNA samples were extracted from total blood of infertile and fertile males using salting out DNA extraction method (32). PCR amplifications were performed using three primer pairs (Table I). Three PCR fragments were amplified as a 557bp fragment from PRM1 gene, a 599bp fragment from PRM2 gene and a 473bp fragment from TNP2 gene. 

The PCR condition for amplification of PRM1 was as, 32 cycles of denaturation at 94^o^C for 1 min, annealing at 66^o^C for 1 min, and extension at 72^o^C for 1 min. PCR condition for PRM2 was as, 32 cycles of denaturation at 95^o^C for 45 sec, annealing at 70^o^C for 45 sec, and extension at 72^o^C for 30 sec and for TNP2 it was as, 32 cycles of denaturation at 95^o^C for 30 sec, annealing at 57^o^C for 1 min and extension at 72^o^C for 30 sec. The PCR products were electrophoresed using 1.5% agarose gel.

**RFLP Genotyping**

For analysis of PRM1 C321A polymorphism RFLP assay, BstuI restriction endonuclease enzyme was used to recognized CGCG sequence. The enzyme digestion was carried out in 2 hours at 37^o^C that cut 557 bp PCR products contained wild type C allele into 321 and 196 bp fragments. For RFLP assay of PRM2 C248T polymorphism, BsrI restriction endonuclease enzyme was used to recognized CCAGT sequence. 

The enzyme digestion was performed for 2 hours at 65^o^C that digest 599 bp PCR products contained wild type C allele into 400 and 199 bp fragments. For TNP2 T1019G polymorphism, EarI restriction endonuclease enzyme was used to recognized CTCTTC sequence. The digestion reaction was in 2 hours at 37^o^C that digested 473bp PCR products contained altered G allele into 353bp and 120bp fragments. 

For TNP2 G1272C polymorphism, HindIII restriction endonuclease enzyme was used to recognized AAGCTT sequence. This enzyme reaction was set up in 2 hours at 37^o^C that digest 473 bp PCR products contained altered C allele in into 375bp and 95bp fragments. All DNA fragments were separated using 1.5% agarose gel electrophoresis and visualized on UV transilluminatore by ethidium bromide staining. 

**SSCP Genotyping**

SSCP analysis was used to screen for G deletion in the nucleotides 1036 and 1046 in TNP2 PCR products and to identify possible unknown mutations because of its simplicity and applicability (Hayashi 1991). All PCR products from cases and controls were analyzed by SSCP Polyacrylamide Gel Electrophoresis (SSCP-PAGE). For the SSCP analysis, five microliters of PCR products were mixed with 25 microliters of SSCP loading dye (95% formamide, 100 mM NaOH, 0.025% Bromphenol blue, 0.025% xylene cyanol). Samples were denatured for 10 min at 94^o^C, cooled on ice and 10 µl were loaded into the polyacrylamide gels contained 6% polyacrylamide (acrylamide/ bisacrylamide, 39:1), 5% glycerol and 1.0X TBE. 

The electrophoresis was carried out at 40 V, 11 mA, and 4^o^C in 1X TBE buffer 16 hours. Gels were visualized using standard silver staining procedure. Shifted fragments from normal controls were cut and gel purified for sequencing. The homology of new sequences with Genbank database was determined using SeqMan (DNASTAR) software.

**Statistical analysis**

Allele frequencies were calculated for each locus by allele counting. Comparisons of allele frequencies between case and control groups and between oligospermia and azoospermia with control group were determined using a Pearson 2 test by SPSS for windows version 16.0 (Chicago, Illinois) software. All tests were two-tailed and p<0.05 was considered as significant value. 

## Results

**Figure 2:**
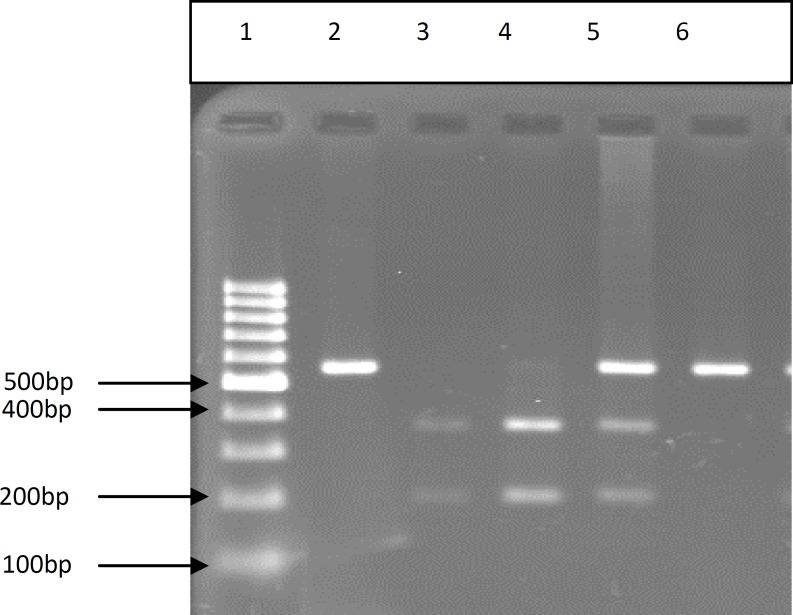
PCR- RFLP of genomic DNA from fertile and infertile men which were analysis for C321A SNP of PRM1. 1) Molecular marker (100 bp), 2) Undigested PCR product for infertile men (AA)(557bp), 3, 4) Completely digested PCR product for fertile men (CC) (361bp, 196bp), 5) Partially digested PCR product for infertile men (CA) p (557bp, 361bp, 196bp), 6) PCR product (control

**Figure 3:**
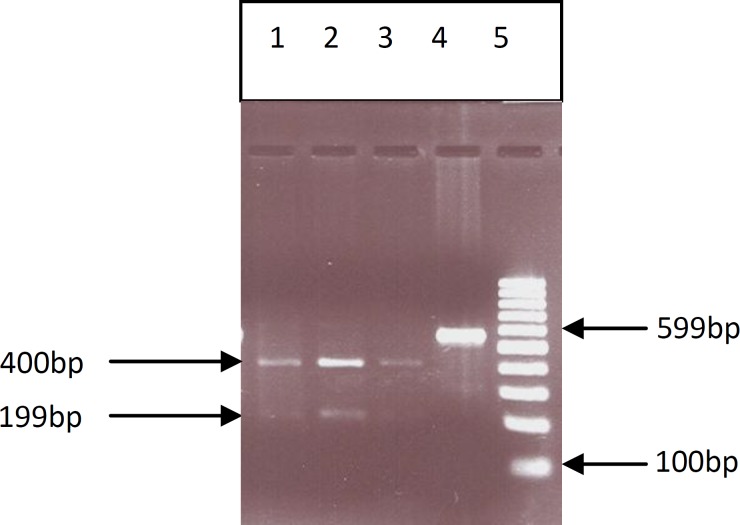
PCR- RFLP of genomic DNA from fertile and infertile men which were analysis for C248T SNP of PRM2 gene. 1, 2, 3) Completely digested PCR product for infertile men (CC)( 400bp,199bp), 4) PCR product (control) (599bp) and 5). Molecular marker (100 bp).

**Figure 4:**
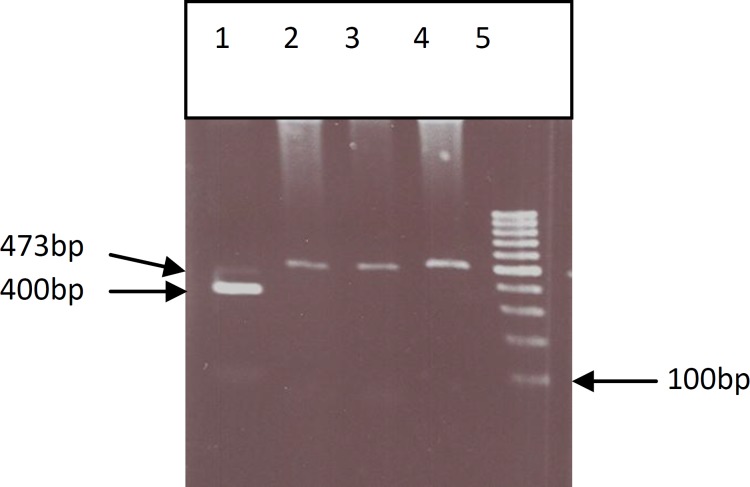
PCR- RFLP of genomic DNA from fertile and infertile men which were analysis for T1019G SNP of TNP2 gene. 1) digested PCR product (control), 2) PCR product (control) , 3, 4) Undigested PCR product for infertile men (TT)(473bp), 5) Molecular marker (100bp).

**Figure 5:**
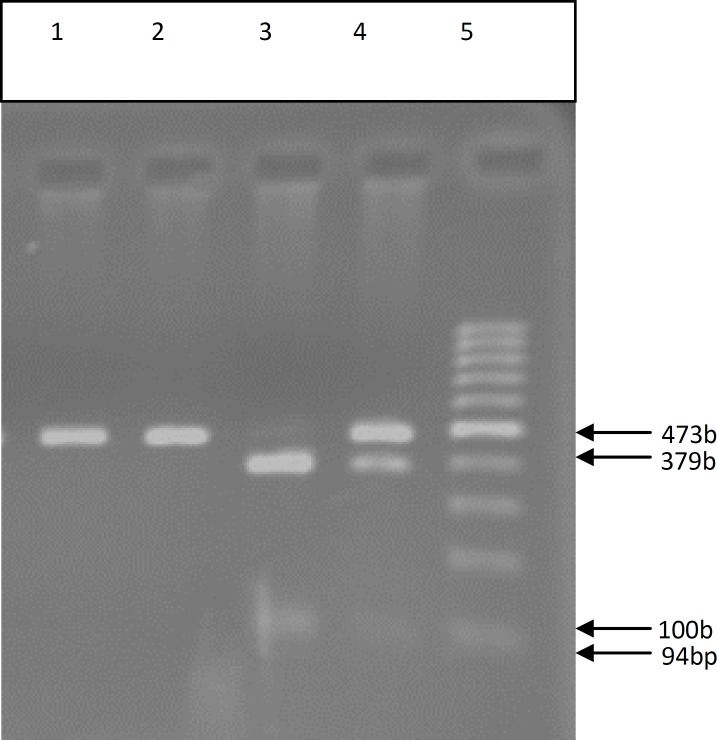
PCR- RFLP of genomic DNA from fertile and infertile men which were analysis for G1272C SNP of TNP2. 1) PCR product (control) (473bp), 2) Undigested PCR product for fertile men (GG) (473bp), 3) Completely digested PCR product for infertile men (CC)(379bp,94bp), 4) Partially digested PCR product for infertile men (GC)(473bp,379bp,94bp), 5) Molecular marker(100bp).

**Figure 6:**
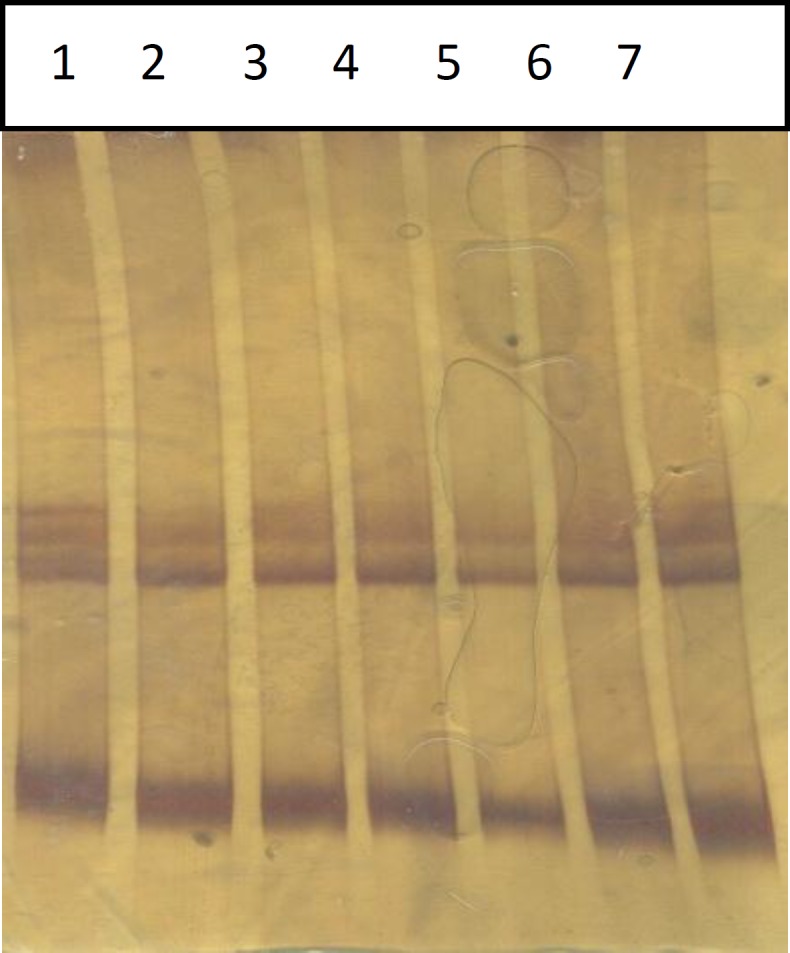
SSCP of PCR products related to TNP2 473bp fragment contained polymorphisms G deletion at nt 1036 and 1046, in infertile men (No 1-5) and normal control (No 6, 7). No polymorphism shifted bond was observed

All SNPs were analyzed in 96 idiopathic infertile men and 100 normal controls (Figure 2-6) (Table II). The first studied SNP was C321A in the PRM1 gene for that the genotype frequencies of the C/C, C/A, and A/A were as 22.5%, 29.9% and 43.6%, in the case group and 24%, 29% and 47% in the control group respectively. 

Although the frequency of altered AA genotype is higher among cases in compare to that in control group but statistical analysis showed no significant difference (p=0.805) between the infertile and fertile Iranian male population (Table II). For the second SNPs, C248T in the PRM2 gene no altered allele was observed among case and control groups in that the genotype frequencies for C/C, C/A, and A/A were as 100%, 0% and 0% in both groups respectively (Table II). 

For the third SNPs in the T1019G of the TNP2 gene no altered allele was observed and the frequencies of the T/T, T/G, and G/G genotypes were 100%, 0% and 0% among both the case and the control groups (Table II). The genotype frequencies for the fourth tested SNPs in the G1272C of the TNP2 genes G/G, G/C and C/C were 51%, 36.5%, and 12.5% whereas, the corresponding frequencies among the control group were 50%, 33% and 17%, respectively. 

However, the frequency of altered allele was higher among normal controls in comparison with the case group and no significant difference (p=0.654) was obtained between infertile and fertile male in Iranian population (Table II). The PCR-SSCP and sequencing of amplified fragment for TNP2 detected single G nucleotide deletion in the 1036 and 1046 nt position of the TNP2 gene in both cases and control groups.

Comparison of alleles frequency of two polymorphic loci in PRM1 (C321A) and TNP2 (G1272C) genes between oligospermic and azoospermic with each other and with control group represented no significant p-values equal to 0.700, 0.847, 0.737 for PRM1 and 0.293, 0.234, 0.838 for TNP2 respectively. It also was not significant when these two polymorphic loci PRM1 (C321A) and TNP2 (G1272C) were considered simultaneously p>0.600 between cases and controls.

**Table I:** Three primer pairs for amplification of the PRM1, PRM2 and TNP2 gene

**Gene**	**Primers**
PRM1	F primer 5'-cccctggcatctataacaggccgc-3' R primer 5'-tcaagaacaaggagagaagagtgg-3'
PRM2	F primer 5'-ctccagggcccactgcagcctcag-3' R primer 5'-gaattgctatggcctcacttggtg-3'
TNP2	F primer 5'-gtggttggtggatgaattggttag-3' R primer 5'-ttctcctttgggtgaaacacgcag-3'

**Table II:** Prevalence of single nucleotide polymorphisms in PRM1, PRM2 and TNP2 in fertile and infertile men

**gene**	**SNP**	**AA** **Change**	**Genotype**	**Fertile** **Number of SNP**	**Allele frequency**	**Infertile** **Number of SNP**	**Allele frequency**	**p-value**
-	C321A	PRM1						
			CC	24 (24%)	C (38.5)	22 (22.5%)	C (38.9)	0.805
			CA	29 (29%)		32 (32.9%)		
			AA	47 (47%)	A (61.5)	42 (43.6%)	A (61.1)	
PRM2	C248T	Glu-Tag						
			CC	100 (100%)	C (100)	100 (100%)	C (100)	0
			CT	0		0		
			TT	0	T (0)	0	T (0)	
TNP2	T1019G	-						
			TT	100 (100%)	C (100)	100 (100%)	C (100)	0
			TG	0		0		
			GG	0	T (0)	0	T (0)	
TNP2	G1272C	-						
			GG	50 (50%)	G (67)	49 (51%)	G (68.7)	0.654
			GC	33 (33%)		35 (36.6%)		
			CC	17 (17%)	C (33)	12 (12.5%)	C (31.3)	
TNP2	G deletion at nt 1036	-						
			GG	100 (100%)	C (100)	100 (100%)	C (100)	0
			G/-	0		0		
			-/-	0	T (0)	0	T (0)	
TNP2	G deletion at nt 1046	-						
			GG	100 (100%)	C (100)	100 (100%)	C (100)	0
			G/-	0		0		
			-/-	0	T (0)	0	T (0)	
Total				100		96		

## Discussion

Recent studies on the variations in human protamine genes in different populations have indicated different speculations (1, 3, 9, 14, 18, 25, -). Some investigations presented a significant relationship between reduced protamine and transition protein 2 gene expressions and male infertility (1, 4, 28, 30). Some SNPs such as C248T alteration in the PRM2 gene have reported in some populations which have been important in male infertility (4). 

This study has evaluated frequency of six previously reported variations in protamine genes cluster consisted of reported mutations in PRM1, PRM2 and TNP2 and their relationship to male infertility in Iranian population. No significant association were observed among two SNPs, C321A in the PRM1 (p=0.805), C248T in the PRM2 (p=0), and four SNPs in the TNP2 consisted of C1019T (p=0), G1272C (p=0.654) and G-del 1036 and 1046 (p=0) in 96 infertile males compared to that in 100 normal male controls. Comparison of allele frequencies of two polymorphic loci in PRM1 (C321A) and TNP2 (G1272C) genes between oligospermic and azoospermic with each other and with control group showed no significant results. It also was not significant when these two polymorphic loci were considered simultaneously p>0.600.

Some rare SNPs of the PRM1 gene were reported to be associated with male infertility, for instance, the G107C point mutation has been observed in 135 case out of 281 infertile men by Imken *et al* 2009 in Moroccan and France and the C190A substitutions was reported to be associated with male infertility, observed in 48.2% of 220 infertile men in Spanish populations and G197T point mutation observed in 10% of 30 infertile men in US population (1, 30). However, in other reported cases for instance, for G107C, was not observed in 672 infertile men in Island by Kichine *et al* 2008 and C321A that was studied by Tanaka *et al* 2003, indicated similar prevalence for infertile and fertile group in Japanese population (4, 34).

Two mutations of C248T and C67T were identified in PRM2 gene in two different studies in infertile cases with azoospermia. First mutation was reported from Japanese by Tanaka *et al* in 2003 and second mutation was reported from Moroccan populations by Imken *et al* 2009 after genotyping of 135 infertile men in both populations. However, G398C mutation in PRM2 gene was also studied in 135 infertile men in Moroccan and 226 infertile Japanese men with no significant difference between infertile and fertile men in both populations (4, 9).

For TNP2 gene, several SNPs have been listed in NCBI. From them, some such as six SNPs, T76G, G117C, C129A, C301T, C391T, C78C in TNP2 gene were studied in 135 infertile men in Moroccan and France populations with no significant difference (9). Two SNPs, T1019G and G1272C in TNP2 gene were also studied by Miyagawa *et al* 2005 in 282 Japanese infertile men but with no significant difference between infertile and fertile populations. In addition, some studies identified the linked genes in protamine cluster (PRM1, PRM2 and TNP2) are expressed specifically in haploid spermatogenic cells in mammals and rare variants in these genes may be potentially have a significant effect on male infertility due to altering gene expression or modifying RNA transcripts in spermatogenesis (12, 28). 

This result showed that the protamine genes were population specific variants and highly conserved during normal male fertilization. Hence, protamine cluster mutations or SNPs that associated with infertility may be infrequent, considering the result from this research and previous studies that indicated that protamine SNPs were not a frequent cause of male infertility. Therefore, tested SNPs in this study could not affect spermatogenesis and were not associated with caused oligospermia and azoospermia in idiopathic male infertility in Iranian population.
